# When Do Objects Become Landmarks? A VR Study of the Effect of Task Relevance on Spatial Memory

**DOI:** 10.1371/journal.pone.0035940

**Published:** 2012-05-07

**Authors:** Xue Han, Patrick Byrne, Michael Kahana, Suzanna Becker

**Affiliations:** 1 Department of Psychology, Neuroscience and Behaviour, McMaster University, Hamilton, Ontario, Canada; 2 Centre for Vision Research, York University, Toronto, Ontario, Canada; 3 Department of Psychology, University of Pennsylvania, Philadelphia, Pennsylvania, United States of America; 4 Department of Psychology, Neuroscience and Behaviour, McMaster University, Hamilton, Ontario, Canada; Goldsmiths, University of London, United Kingdom

## Abstract

We investigated how objects come to serve as landmarks in spatial memory, and more specifically how they form part of an allocentric cognitive map. Participants performing a virtual driving task incidentally learned the layout of a virtual town and locations of objects in that town. They were subsequently tested on their spatial and recognition memory for the objects. To assess whether the objects were encoded allocentrically we examined pointing consistency across tested viewpoints. In three experiments, we found that spatial memory for objects at navigationally relevant locations was more consistent across tested viewpoints, particularly when participants had more limited experience of the environment. When participants’ attention was focused on the appearance of objects, the navigational relevance effect was eliminated, whereas when their attention was focused on objects’ locations, this effect was enhanced, supporting the hypothesis that when objects are processed in the service of navigation, rather than merely being viewed as objects, they engage qualitatively distinct attentional systems and are incorporated into an allocentric spatial representation. The results are consistent with evidence from the neuroimaging literature that when objects are relevant to navigation, they not only engage the ventral “object processing stream”, but also the dorsal stream and medial temporal lobe memory system classically associated with allocentric spatial memory.

## Introduction

In everyday life, an object may be attended to individually, or may be processed within the spatial context of a scene. Traditionally these two styles of processing are associated with two major branches of the visual system, the ventral “what” stream and dorsal “where” or “how to” stream [1]–[3]. More specifically, when viewed within its spatial context, a single object could be encoded either within a viewpoint-dependent (egocentric) representation, e.g. as a visual snapshot memory, or within a world-centered or allocentric frame of reference. Accordingly, the notion of a single dorsal visual stream for spatial processing has been updated to include three branches: a parieto-prefrontal branch involved in visuospatial working memory, a parieto-premotor branch involved in visually guided action and a parieto-medial temporal branch involved in spatial navigation [1]. When there are multiple objects, the possibilities are even greater. A collection of objects could be treated as a whole entity and encoded as one configuration, either as an egocentric/view-based snapshot or allocentrically based on inter-object relations. Many studies have tested memory for sets of objects on rotating tabletops to investigate this type of encoding (e.g. [4]). Alternatively, each of the objects could be encoded allocentrically with respect to features of the environment such as buildings or geometric cues. Finally, as we argue here, the brain may employ simultaneously a hierarchy of representations, from egocentric representations of sensory information to allocentric representations in long-term memory. This could allow some egocentric cues, such as a familiar viewpoint or a dominant reference direction within an environment, to have preferential access into allocentric long-term memory.

Some early empirical studies led to rather polarized views on how objects are encoded. For example, memory for object locations can be disrupted by disorientation (e.g. [5]), and is best when the tested viewpoint (imagined heading) is congruent with one of the studied viewpoints [6], [7]. These data seem inconsistent with an orientation-invariant representation of object configurations. We return to this issue later in the introduction, where we discuss the use of combined egocentric and allocentric representations, as in the BBB model. On the other hand, if one learns the environment by directly experiencing it from multiple perspectives, as opposed to by studying a map, spatial memory of the relation between items is more robust to viewpoint rotations, suggesting allocentric encoding of objects [8]. Whereas viewpoint-invariance could simply arise from storing multiple view-based snapshots, strong neurobiological support for allocentric representations comes from evidence of place cells – neurons that respond selectively when an animal is in a given location. Such cells, which have been identified in the hippocampi of rats [9], non-human primates [10] and humans [11], are often insensitive to the animal’s heading within the environment, suggesting that they encode spatial location within an allocentric representation. Moreover, hippocampal damage impairs allocentric memory function. For example, an individual who suffered perinatal hippocampal pathology showed highly impaired memory for arrays of objects when tested from unfamiliar viewpoints, in spite of highly accurate memory when tested from familiar viewpoints [12].

The mixed evidence in support of egocentric vs. allocentric representations likely reflects people’s ability to use both types of representation. Methodological differences such as passive versus active navigation and exposure to few versus multiple viewpoints may contribute to the type of processing people engage in. Participants in the Evans et al. [8] and King et al. [12] studies learned the environment by active navigation, whereas those in the Roskos-Ewoldsen et al. [6] and Shelton et al. [7] studies learned the environment from one or two static views. Thus, active navigation, and/or exposure to a dynamically changing range of views of the environment, may encourage allocentric strategies. Consistent with this notion, rodent place cells tend to be omnidirectional when recorded in the open field but unidirectional when recorded in a linear track or narrow-armed mazes [13]–[16]. When humans take a path around the square road in a virtual environment one observes both unidirectional place cells (as in the rat) and also path cells that are sensitive to the direction of motion independent of the (virtual) location within the environment [17].

An emerging view is that allocentric and egocentric representations coexist and recruit different levels of representation [18]. If we accept that incoming visual input is by definition egocentric (i.e. retinocentric), and that we have the capacity to create allocentric representations (e.g.hippocampal place cells), it follows that allocentric representations of the world can only be constructed from egocentric inputs. Thus, when we encode information, we have the option of employing a purely egocentric strategy or a combined strategy that includes multiple levels, mapping from egocentric to allocentric frameworks. It is likely that we have developed specialized circuits that may be predominantly egocentric, or may also include allocentric representations. This is supported by a wide range of evidence from behavioural, neuroimaging and brain lesion studies in humans and other animal species (e.g. [19]–[26]). These two types representations are differentially governed by the traditional rules of associative learning (e.g. blocking and overshadowing) [27] and vary according to task demands (e.g. [4], [24], [25]. Even when performance is behaviourally equivalent, when people employ allocentric representations they activate distinct neural circuits [22]. Thus, wayfinding and other allocentric spatial tasks recruit a common neural circuit including the parietal cortex, retrosplenial cortex, fusiform gyrus, precuneus, parahippocampal gyrus, hippocampal complex and several prefrontal cortical regions, while non-spatial navigation tasks such as learning a series of body turns recruit an associative learning circuit involving the striatum (including the caudate nucleus and putamen), insula/ventrolateral prefrontal cortex, and right anterior prefrontal cortex (e.g. [22], [28]–[31]).

Given the abundant evidence for allocentric representations, an important question is how allocentric representations could arise out of purely egocentric (i.e. retinocentric) sensory input. Byrne, Becker, and Burgess [32] proposed a computational model, which we shall refer to as the BBB model, suggesting that egocentric information about the spatial locations of objects from the dorsal visual pathway is combined with object appearance information from the ventral visual pathway to form allocentric, configural representations of spatial environments in long-term memory at the level of the hippocampus. Conversely, memories about spatial configurations can be retrieved from (allocentric) long-term memory in the hippocampus and mapped through reciprocal neuronal pathways to generate egocentric mental images. Note that individual objects may also be represented allocentrically within the ventral visual pathway, e.g., there is evidence for view-invariant representations of single objects within inferotemporal cortex [33]; this type of object-based allocentric representation must be distinguished from the *configural* allocentric representations of scenes referred to here, mediated by the medial temporal lobe. Because the BBB model postulates that egocentric level representations provide access cues to allocentric long-term memory, it naturally accommodates preferred viewpoint effects, for example, as defined by intrinsic frames of reference formed from egocentric experiences and environmental cues [4], [34]–[36]. Note, however, that the BBB model does not incorporate the non-spatial associative learning circuit mentioned above. The role of the dorsal visual pathway in the BBB model encompasses both the parieto-medial temporal branch [1] for forming allocentric representations, and the parieto-frontal branch [1], for maintaining and updating object locations in working memory after real or imagined observer motion.

The BBB model postulates some of the neural mechanisms that may underlie allocentric spatial memory, but it does not tell us what sort of features might contribute to the creation of these memories. As mentioned above, one important factor that may contribute to allocentric coding of features is their utility for spatial memory and navigation. For example, objects placed at choice points should be particularly relevant to navigation. Several experiments have examined the impact of navigational relevance on object recognition memory. Janzen and van Turennout [37] had participants passively view a movie of a tour through a virtual museum with objects placed at T-shaped intersections (decision points) and simple L-shaped turns (non-decision points), and directed their attention more to some of the objects (toys) than others, half of which were placed at decision points. Although both types of locations lead to a change in one’s direction, and as such, could both be considered as decision points (e.g. [38]), we adhere to the terminology as used by Janzen & van Turrenout throughout this manuscript. While recognition memory accuracy was not affected by navigational relevance or attention, reaction times were faster for attended objects (toys), and fastest of all to the toys that had been seen at decision points. Moreover, functional imaging studies show greater activation of medial temporal and medial parietal structures associated with spatial cognition (including the hippocampus, parahippocampal cortex, superior parietal lobule/precuneus, parietal-occipital sulcus, retrosplenial/anterior calcarine region) for navigationally relevant objects in recognition memory [37] and object priming tasks [38], [39], and also greater activation in these regions when encoding virtual environments containing landmarks (salient objects) compared to encoding a plain empty virtual environment [30]. These studies suggest that objects are not always just objects: when they are relevant to navigation, they are much more likely to recruit allocentric spatial memory circuits. One potential confound with the above studies on navigational relevance is that objects at decision points may be inherently more salient. Furthermore, they assessed recognition memory and priming, but not spatial memory. Miller and Carlson [40] used a setup similar to Janzen and van Turennout's [37] with an explicit manipulation of object salience, and measured both recognition memory and spatial memory (map drawing). They found that spatial memory for decision-point objects was still superior even when they were less salient than non-decision-point objects, whereas recognition memory was strongly modulated by salience. Thus navigational relevance seems to strongly modulate whether objects are incorporated into spatial memories.

The studies reviewed above suggest that 1) people use both egocentric and allocentric strategies for spatial memory and navigation, depending on task demands, and 2) the hippocampal and parahippocampal regions are crucial for allocentric spatial memory formation and are recruited for encoding objects that are relevant to navigation. It remains to be demonstrated whether navigational relevance causes a switch in favor of allocentric encoding of objects. Thus, the experiments reported here were designed to test the hypothesis that navigational relevance would modulate the degree to which objects would be integrated within their spatial context into allocentric spatial maps. To assess the degree of viewpoint invariance of object memory, we developed a novel VR pointing task and a novel performance metric – pointing consistency across tested viewpoints.

We conducted three experiments to test whether objects would be encoded differently based on navigational relevance, and whether the type of attention paid to objects would modulate this effect. Whereas Janzen and van Turennout [37] and Miller and Carlson [40] had participants passively view image sequences of a virtual environment, we wanted a more life-like task where people actively control where they go, how long they spend in each location, and what they pay attention to. They should thereby construct an internal representation of an environment using whatever features are most relevant to navigation and spatial orienting. We constructed a set of virtual towns with grid-like streets lined with stores using Kahana’s “Yellow Cab” virtual taxicab simulator (http://memory.psych.upenn.edu/Research). Using this same task, in human intra-cranial recordings, Ekstrom and colleagues [11] found evidence of place cells and view cells in the human medial temporal lobe, indicating that even this relatively simplistic task and artificial environment engages the standard spatial memory circuits and evokes allocentic spatial representations (see, also, Jacobs et al. [17]). We asked participants to pretend to be a taxi driver in the town and look for and deliver passengers. We placed objects at certain locations in the town, half at decision points (T-shaped intersections) and half at non-decision points (L-shaped intersections). Participants implicitly learned the stores and object locations by playing the taxi game, and were then given tests of recognition memory and spatial memory for the objects after each of the study phases. In spatial memory test trials, memory for the locations of the objects was probed from two different viewpoints, which were views of the town from the two end-points, marked by “Mike’s Restaurant” and “House of Pizza” respectively.

In Experiment 1, participants learned the layout of the virtual town via active navigation, while pretending they were taxi drivers looking for and delivering passengers. In Experiment 2, participants learned the town layout passively by watching videos of trajectories through a town. We also included a between-subjects manipulation in Experiments 1 and 2 to vary the number of starting points that participants would experience. In our study, Experiments 1 and 2 each had two conditions, one in which participants started navigation trials alternatingly from two points, creating two salient viewpoints/reference directions from which spatial memory could be accessed, and one in which participants always started from the same point, creating one salient viewpoint/reference direction [34], [41], [42]. Consistent with previous research by McNamara and colleagues, we expected that participants’ spatial memory would be superior when tested from the most salient viewpoint when they always started from the same end of the town. We also hypothesized that having experienced the town from two different starting points, participants would tend to approach the objects from multiple directions and would thus be more likely to form view-invariant representations of those objects.

One potential confound in Experiments 1 and 2 is that objects at decision points may be attended to more strongly or for more time than objects at other locations. Thus even if memory for objects at decision points is superior, it does not necessarily mean those objects were processed via a different neural circuit or a qualitatively different mechanism. To address this issue, in Experiment 3, we explicitly manipulated the type of attention devoted to objects, by instructing participants to only focus on either the appearance or the location of objects. We hypothesized that the type of attention would modulate the effect of navigational relevance, that is, memory for objects at decision points should benefit from spatial attention and should be hurt by attention to object appearance.

A key issue in the present study is how best to assess the degree of viewpoint invariance of participants’ spatial memory for objects. Most previous research in object spatial memory has employed small rooms within which all objects could be viewed from a single location (e.g. [5], [34] or a rotating tabletop upon which the entire configuration of objects could be viewed simultaneously (e.g. [4]). In these studies, various measures of memory for object configurations have been employed, such as “configuration error” [5] and judgments of relative direction (e.g. (“Imagine you are at the A and facing the B. Point to the D.”) (e.g. [34], [36]). These measures of errors in memory for inter-object relations are suitable for testing hypotheses about memory for object configurations, but do not address our main question of whether objects are encoded relative to environmental and geometric cues. In our experiments, we use large virtual towns, with streets lined with buildings and shops, and objects located all around the town. Thus, in our experiments, the objects could not be directly perceived as a configuration within a single location, but would have to be learned individually by actively navigating in the town or watching video tours of the town, integrating the information over larger spatial and temporal extents. We thus expected participants would encode each object relative to the surrounding visible environmental features. We used a novel method to assess viewpoint invariance of spatial memory for objects across different locations within the environment. We calculated the consistency of pointing responses to each object (see Method section) made from two different viewpoints at opposite ends of the town. We reasoned that if participants were encoding object locations relative to an allocentric spatial map of the town, they should make consistent pointing errors when tested from either viewpoint. For example, if an object was in the middle of the town and they mis-localized it by 45 degrees clockwise when pointing from one end of the town, they should mis-localize it by about the same amount and in the opposite direction, 45 degrees counterclockwise, when pointing to it from the opposite end of town. Therefore, we developed a measure of pointing consistency across tested viewpoints. We acknowledge that accuracy for accessing spatial memories from perspectives 180 degrees from the stored perspective is better than from other perspectives (e.g. 45 degrees or 135 degrees), however, it is still worse than accessing it directly from the stored perspective (e.g. [25], [34], [43]). Furthermore, in large-scale environments, accessing from the opposite direction of the stored perspective was found to be no easier than from other directions [26].

To summarize our predictions, we hypothesized that 1) objects at more navigationally relevant locations (decision points) should be encoded as landmarks, and become incorporated within an internal cognitive map of space. Although access to this internal representation via egocentric cues could be biased along a preferred orientation (as per Valiquette, et al. [43]), the navigational relevance of objects within the environment should still modulate the degree to which their internal representation is sensitive to changes in viewpoint. 2) When participants experienced the town from fewer viewpoints they should be even less likely to employ allocentric strategies for objects, particularly those at locations not relevant to navigation. Thus, reducing the number of starting points should reduce the number of familiar viewpoints, and thereby enhance the effect of navigational relevance. 3) When participants’ attention was manipulated to focus on objects’ appearance, the decision-point effect would be eliminated, whereas when participants’ attention was directed toward objects' locations, this effect would persist or even be enhanced.

## Results

### Experiment 1

In Experiment 1, participants implicitly learned the town layout and object locations by playing a virtual taxi game requiring active navigation through a virtual town. We varied the navigational relevance of objects in the environment by placing them either at decision points or non-decision points. In Condition A, participants started passenger pickups alternatingly from the two ends of the town marked by House of Pizza and Mike’s Restaurant respectively, and were subsequently tested from both of those viewpoints. This would establish two salient viewpoints/reference directions, which were also the tested viewpoints, from which either type of object could be encoded. In Condition B, participants entered the town from only one direction, facing the House of Pizza, thereby establishing only a single salient viewpoint/reference direction during study. Nevertheless, in both conditions, we tested participants’ memory from the same two viewpoints, one facing House of Pizza and the other facing Mike’s Restaurant. In Condition B, by always having the participants start navigating from one end of town rather than two, we introduced an encoding bias. If indeed decision-point objects were encoded as part of an allocentric map of the town whereas non-decision-point objects were not, spatial memories for decision-point objects should be less affected by this manipulation relative to other objects. Therefore, we predicted that the pointing responses would be less accurate and less consistent for non-decision-point objects in Condition B relative to those in Condition A, but memory for decision-point objects should be similar across the two conditions, if objects at decision points were encoded as part of an allocentric map, relative to other objects. Moreover, when participants experienced the two tested viewpoints equally, the difference between decision points and non-decision points would be reduced.

#### Recognition accuracy

Recognition memory was better for decision-point objects and it was better when there was a single starting point. A two-way repeated measures Place (decision point vs. non-decision point) x Condition (one starting point vs. two) ANOVA revealed significant main effects of Place [*F* (1,58) = 8.706, *p* = 0.005] and Condition [*F* (1,58) = 5.342, *p* = 0.024], but no interaction between Place and Condition [*F* (1,58) = 0.455, *p* = 0.503]. Recognition accuracy was significantly better for objects at decision points (*mean* = 90.7%, *SE* = 0.011) than for those at non-decision points (*mean* = 87.1%, *SE* = 0.013) across conditions. Unexpectedly, recognition memory was also significantly better when participants used one starting point (Condition B *mean* = 91.4%, *SE* = 0.015) than two (Condition A: *mean* = 86.4%, *SE* = 0.015).

#### Pointing latency

Pointing latency was faster when there was a single starting point, but it was not affected by navigational relevance or viewpoint. A three-way repeated measures Place (decision points vs. non-decision points) x Condition (one starting point vs. two) x tested Viewpoint (Mike’s Restaurant vs. House of Pizza) ANOVA of the pointing/recognition latencies revealed a significant main effect of Condition [*F* (1,58) = 5.705, *p* = 0.02], but no significant effect of Place [*F* (1,58) = 0.024, *p* = 0.877] or tested Viewpoint [*F* (1,58) = 1.388, *p* = 0.244] and no significant interactions. Responses were significantly faster in Condition B (one starting point) (*mean* = 4.578, *SE* = 0.409) than in Condition A (two starting points) (*mean* = 5.959, *SE* = 0.409) across object types and tested viewpoints.

#### Pointing errors

Navigational relevance affected pointing accuracy when there was a single starting point. A three-way repeated measures Place x Condition x tested Viewpoint ANOVA of the pointing errors revealed significant main effects of Place *[F (1,58) = 6.751, p = 0.012]* and tested Viewpoint [*F* (1, 58) = 7.369, *p* = 0.009], and significant interactions between Place and Condition [*F* (1, 58) = 5.964, *p* = 0.018] and between tested Viewpoint and Condition [*F* (1,58) = 14.275, *p* < 0.001], but no main effect of Condition [*F* (1,58) = 3.047, *p* = 0.086] alone and no other significant interactions, see Table 1. Pointing errors were significantly smaller for objects at decision points (*mean* = 26.479, *SE* = 1.254) than those at non-decision points (*mean* = 28.239, *SE* = 1.441) across conditions and pointing errors were significantly smaller when they were made from House of Pizza viewpoint (the starting point in Condition B, *mean* = 26.043, *SE* = 1.295) than when they were made from the Mike's Restaurant viewpoint (*mean* = 28.675, *SE* = 1.487) across both types of object and both conditions (one start point or two).

**Table 1:** **Table 1.** Average pointing errors in degrees for experiments 1, 2 and 3.

Tested Viewpoint	Place	Expt. 1-A Two starting points	Expt. 1-B One starting point (HoP)	Expt. 2-A Two ways	Expt. 2-B One way(Mike)	Expt. 3- Appearance (HoP)	Expt. 3-Location (HoP)
House of Pizza	DPs	25.41 (1.80)	25.05 (1.81)	30.11 (1.84)	31.93 (2.50)	31.85 (4.00)	32.79 (4.07)
	NDPs	25.78 (1.92)	27.94 (2.21)	30.42 (1.63)	32.06 (2.38)	28.03 (3.83)	29.28 (3.90)
Mike’s Restaurant	DPs	24.64 (1.84)	30.82 (2.15)	31.81 (2.07)	28.30 (2.32)	33.48 (3.69)	33.79 (3.75)
	NDPs	24.48 (2.22)	34.76 (2.56)	32.59 (1.92)	29.78 (2.26)	31.30 (3.70)	39.20 (3.77)

DPs represent decision points; NDPs represent non-decision points; numbers in the bracket represent standard errors; HoP represents that House of Pizza is the starting point and Mike represents that Mike’s Restaurant is the starting point.

To further investigate these significant interactions, we conducted separate two-way repeated measures Place x tested Viewpoint ANOVAs for the two conditions. In Condition A, when participants started alternatingly from both ends of the town during study, there was no main effect of Place [*F* (1, 29) = 0.013, *p* = 0.911] or tested Viewpoint [*F* (1, 29) = 1.012, *p* = 0.323], and no interaction between Place and tested Viewpoint [*F* (1, 29) = 0.065, *p* = 0.801]. In Condition B, when participants always started from the same end of the town, there were significant main effects of Place [*F* (1, 29) = 12.082, *p* = 0.002] and tested Viewpoint [*F* (1, 29) = 14.625, *p* = 0.001], but no interaction between Place and tested Viewpoint [*F* (1, 29) = 0.521, *p* = 0.476]. Pointing errors were significantly smaller for objects at decision points (*mean* = 27.934, *SE* = 1.822) than for objects at non-decision points (*mean* = 31.349, *SE* = 2.171) across viewpoints and pointing errors were significantly smaller when they were made from House of Pizza viewpoint (the starting point, *mean* = 26.493, *SE* = 1.935) than when they were made from the less familiar Mike's Restaurant viewpoint (*mean* = 32.79, *SE* = 2.272) across object types in Condition B.

#### Pointing consistency: Standard deviations

**Figure 1:**
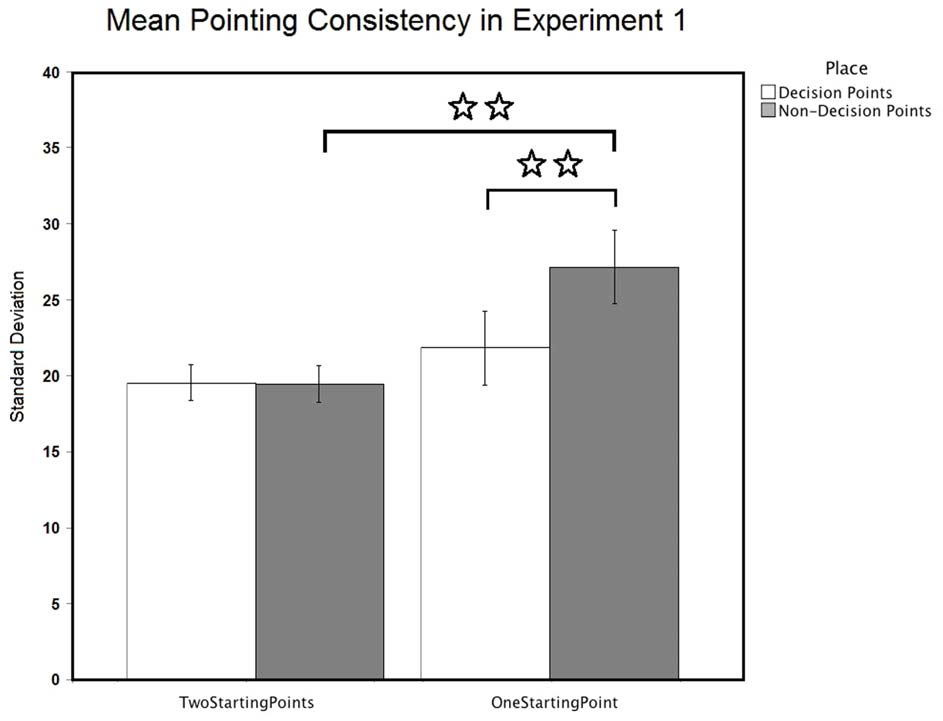
Figure 1. Pointing consistency in Experiment 1. Mean pointing consistency scores (95% confidence intervals) for decision and non-decision-point objects in Experiments 1-Active navigation (two starting points vs. one starting point). White bar is for decision points and grey bar is for non-decision points. The pointing responses were significantly more consistent for objects at decision points than for those at non-decision points in Experiment 1b (one starting point), but not in Experiment 1a (two starting points). ⋆⋆ means *p*<0.01.

Navigational relevance affected pointing consistency when there was a single starting point. A two-way repeated measures Place x Condition ANOVA of pointing consistency scores revealed significant main effects of Place [*F* (1, 58) = 7.794, *p* = 0.007] and Condition [*F* (1, 58) = 4.964, *p* = 0.03] and a significant interaction between Place and Condition [*F* (1, 58) = 8.264, *p* = 0.006]. Pointing responses were significantly more consistent for objects at decision points (*mean* = 20.659, *SE* = 1.013) than for those at non-decision points (*mean* = 23.272, *SE* = 1.37) across conditions, and were significantly more consistent in Condition A (*mean* = 19.491, *SE* = 1.571) than in Condition B (*mean* = 24.44, *SE* = 1.571) across object types. To further investigate the interaction between Place and Condition in terms of pointing consistency, two-tailed paired sample *t*-tests were used. In Condition A, there was no difference in pointing consistency by object location (*t* = 0.095, *df* = 29, *p* = 0.925). In Condition B, pointing responses were significantly more consistent at decision points than at non-decision points (*t* = −3.147, *df* = 29, *p* = 0.004). Moreover, because we hypothesized that reducing the number of starting points (Condition B) would reduce pointing consistencies for objects at non-decision points. Two-tailed independent *t*-tests showed that pointing consistencies for objects at decision points were no different between the two conditions (*t* = −1.115, *df* = 56.21, *p* = 0.27), but significantly worse for objects at non-decision points (*t* = −2.787, *df* = 49.006, *p* = 0.008) in Condition B than Condition A (Note: controlling for multiple comparisons, significant *p* value is 0.0125; Equal variances were not assumed). The analysis revealed that the navigational relevance effect was only significant in the single-starting-point condition (Condition B), in which pointing responses were more consistent for objects at decision points than for those at non-decision points, but not in the two-starting-point condition (Condition A). Moreover, reducing the number of starting points during the study phase detrimentally affected the pointing consistencies for objects at non-decision points, but had little effect on objects at decision points, see Figure 1.

#### Pointing consistency: correlation between two tested viewpoints

One reason pointing responses were more consistent (i.e. less variable) across viewpoints in the case of objects at decision points could simply be that the pointing errors themselves were smaller for decision-point objects. Even if the pointing responses from the two viewpoints were uncorrelated, smaller magnitude pointing errors would lead to smaller standard deviations in pointing errors. To rule out this possibility, we also employed a secondary measure of pointing consistency that is insensitive to overall error magnitude: The Pearson correlation coefficient (*r*) between the two signed pointing errors at the two tested viewpoints across blocks was calculated for each type of object. Therefore, each participant had one Pearson’s *r* for decision-point objects and one for non-decision-point objects. Because we hypothesized that the decision-point objects would be less affected by viewpoint changes, these correlation scores were compared using one-tailed nonparametric Wilcoxon Signed Rank tests, which revealed that signed pointing errors were significantly more correlated for objects at decision points (*mean r* = 0.377, *SE* = 0.052) than those at non-decision-point objects (*mean r* = 0.213, *SE* = 0.068) (*p* = 0.014) in Condition B (one starting point), but not in Condition A (decision points *mean r* = 0.326, *SE* = 0.057; non-decision-point objects *mean r* = 0.402, *SE* = 0.055) (*p* = 0.1495) (Note: controlling for multiple comparisons, significant *p* value is 0.025) Thus, the correlation analysis was in complete agreement with our standard deviation measure of pointing consistency, indicating that memory for decision-point objects in Condition B was more view-invariant, and not just more accurate.

#### Consistency of signs of pointing errors

Another limitation of our pointing consistency measure is that it is sensitive to the locations of the objects in the town, such that if an object was closer to one end of the town than the other, even if the participant consistently mis-localized it to the same location from both ends of the town, the angular error magnitudes would differ. This is not a confound, because it is equally true for both decision and non-decision-point objects. However, an alternative measure that is insensitive to the angular error magnitude is the consistency of the signs of the errors. If a participant consistently mis-localizes an object, for example, clockwise from one end and counterclockwise from the other end, the signs of the errors would be consistent. Note: The signs of the pointing errors from one end of town were reversed; see Method-Exeperiment 1-Data Analysis-Pointing Error (Average Absolute Pointing Errors) for details. There were 4 pairs of pointing responses made for 4 decision-point objects and another 4 for the four non-decision-point objects in each block, if the participant correctly identified all of the objects, and there were 4 blocks. We calculated the percentage of pairs of pointing errors that had the same sign over blocks for decision-point objects and then for non-decision-point objects. Because we hypothesized that the decision-point objects would be less affected by viewpoint changes, one-tailed Wilcoxon Signed Rank tests were used revealing that the percentage of same signed pointing errors for decision points was significantly higher than those for the non-decision-point objects in Condition B (*p* = 0.0135, DPs mean = 66.93%, SE = 0.033; NDPs mean = 58.14%, SE = 0.032), but not in Condition A (*p* = 0.457, DPs mean = 62.88%, SE = 0.035; NDPs mean = 62.26%, SE = 0.035) (Note: controlling for multiple comparisons, significant *p* value is 0.025). Thus, the analysis of consistency of signs of pointing errors was in complete agreement with our standard deviation measure of pointing consistency, indicating that memory for decision-point objects in Condition B was more view-invariant, and not just more accurate.

#### View time

View Time was longer for objects at decision points across conditions. A two-way repeated measures Condition × Place ANOVA of view time revealed a significant main effect of Place [*F* (1, 58) = 146.56, *p*<0.0001] and a significant interaction between Place and Condition [*F* (1, 58) = 7.571, *p* = 0.008], but no main effect of Condition [*F* (1, 58) = 0.021, *p* = 0.885]. Viewing time for objects at decision points (*mean* = 29.9%, *SE* = 0.004) was longer than for objects at non-decision points (*mean* = 21.4%, *SE* = 0.005) across conditions (Condition A: DP *mean* = 28.9%, *SE* = 0.006, NDP *mean* = 22.3%, *SE* = 0.007; Condition B: DP *mean* = 30.9%, *SE* = 0.006, NDP *mean* = 20.5%, *SE* = 0.007). To investigate the interaction between Place and Condition, two-tailed paired sample *t*-tests were conducted, which revealed that viewing time for decision-point objects was significantly longer than for non-decision-point objects in both conditions (*p*s<0.001).

#### View time: correlation between view time and other spatial measurements

View time was not correlated with spatial memory accuracy or consistency. Given the significant difference in viewing time between objects at decision points and non-decision points, any potential differences we might observe in spatial memory for these objects in the current experiment could be due to more attention and encoding time being devoted to decision-point objects (a potential confound). Alternatively, the viewing time differences may be entirely due to participants engaging other processes at decision points, such as imagining the route along alternate paths and making navigation decisions. While the lack of spatial memory differences between the two types of objects in Condition A (two starting points) suggests the latter interpretation, viewing time differences could still be a potential confound in Condition B (one starting point). If the reason participants spent more time viewing decision-point objects was partly due to greater time devoted to attending to and encoding those objects’ locations, we would expect viewing time to correlate with memory for those objects. We therefore assessed whether any of the pointing error and consistency measures were correlated with view time for both decision and non-decision points. These correlational analyses revealed that none of our memory measures were significantly correlated with viewing time.

We hypothesized that navigational relevance would strongly modulate whether objects were treated as landmarks and encoded within an allocentric cognitive map, particularly when objects were seen from a limited range of viewpoints. Although participants were free to navigate around the town and potentially approach each object from multiple directions, the single starting point would bias participants to approach each object from fewer directions, on average. This led to our prediction that spatial object memory would be more accurate and more viewpoint-invariant for objects at decision points than for other objects, particularly when we reduced the number of starting points. Our results confirmed this prediction. While the two object types showed differences in viewing time and recognition memory accuracy in both conditions, there was no effect of navigational relevance on any of the spatial memory measures in Condition A, where participants used two different starting points. On the other hand, in Condition B, when there was only one starting point, spatial memory for objects that were not at decision points suffered, such that pointing responses were less accurate and less consistent across the two tested viewpoints. Thus, as predicted, spatial memory for non-decision point objects was sensitive to the number of starting points, whereas spatial memory for decision point objects was less affected.

Interestingly, when participants began navigation from both ends of the town, the navigational relevance effect was not merely diminished but disappeared altogether, see Figure 1. One reason for this lack of effect of navigational relevance in Condition A could be that when experienced from more viewpoints, even objects at “non-decision points”, i.e. L-intersections, come to be treated as landmarks. Although L-intersections are less navigationally relevant than T-intersections, they do involve a turn in the route and are thus more relevant when compared to straight portions of a route. Future studies could investigate this possibility, by including objects along straight roads. Another possibility is that when experienced from both ends of the town, the objects at non-decision points were encoded as multiple egocentric snapshots. In either case, pointing consistency differences between decision and non-decision-point objects would disappear. One way to tease apart these two alternative explanations would be to repeat the fMRI study by Janzen and van Turrenout [37] in which participants viewed a trajectory through a virtual museum containing objects at both decision and non-decision points. However, rather than viewing the tour in one direction only, they could view the tour in both directions as in our Experiment 2. If this caused a switch from egocentric to allocentric/dorsal visual stream encoding for the objects at non-decision points then those objects should now activate the parahippocampal region.

Unexpectedly, the number of starting points also affected pointing response latencies and recognition accuracy, but in the opposite direction to the consistency effects. As mentioned above, pointing errors were more consistent in Condition A, the condition with two starting points. In contrast, pointing responses were faster and recognition memory was more accurate in Condition B, the single starting point condition, across both object types and both tested viewpoints. One possible explanation for these results is that some participants were using a mental navigation strategy to recall object locations. Such a strategy would be fastest when there was a single starting point, and more likely to break down as the number of to-be-remembered routes increased. Individual differences in strategy are often seen in spatial cognition studies, and certainly warrant further investigation in the tasks studied here.

Not surprisingly, we saw an effect of the specific viewpoint in Condition B: In the case of a single starting point at House of Pizza, pointing errors were smaller from the more familiar House of Pizza viewpoint than from Mike’s Restaurant viewpoint for both types of objects. This is consistent with the findings of Mou and Colleagues [4], [34]–[36] reviewed in the introduction, and fits within the BBB model which postulates that egocentric retrieval cues are used to index long-term allocentric memory.

Importantly, in spite of the preferred viewpoint effect on both types of objects, our pointing consistency analysis revealed that spatial memory for the two types of objects was differentially affected by the reduced number of starting points in Condition B (relative to Condition A). Pointing errors were significantly more consistent across the tested viewpoints for objects at decision points than for those at non-decision points, using the standard deviation (pointing consistency), the correlation analysis and the consistency of signs of pointing errors analysis. This finding is consistent with the hypothesis that objects at decision points are more likely to be incorporated within an allocentric map, less affected by the number of salient reference directions, and more robust to changes in viewpoint at test time. It also supports our claim that pointing consistency across tested viewpoints is a useful measure of allocentric coding when objects are seen in large-scale spaces, as opposed to being viewed from a single location.

Another possible explanation for the superior spatial memory for decision-point objects in Condition B is that they were not encoded in a qualitatively different manner, but were simply better encoded than were non-decision-point objects. For example, participants may have devoted more attention to decision-point objects. Consistent with this alternative interpretation, recognition memory was superior and viewing times were longer for these objects. However, it is important to note that our “view time” measure was not a pure measure of the time a participant was actually attending to each object, as it would also include the time spent making navigational decisions. Accordingly, participants often stopped at intersections and looked around before deciding where to go next. More importantly, this alternative explanation cannot account for the lack of significant differences in spatial memory for decision-point and non-decision-point objects in Condition A (two starting points) in spite of equivalent differences in viewing times. Moreover, there was no correlation between viewing time and any of our spatial memory measures.

Although attentional differences between the two types of objects do not seem to be the most likely explanation for the superior memory for decision-point objects in Condition B, we cannot entirely rule out this possibility when participants are freely navigating in the environment and are free to re-visit any location as often as they like. Thus viewing times and experienced viewpoints of each object are not strictly controlled. Moreover, objects at decision points could be seen from three directions, whereas objects at non-decision points only could be seen from two directions, when participants were actively driving in the town. Thus, even when we eliminated one starting point in Condition B, the inherent difference in the number of experienced views for objects at L-shaped versus T-shaped intersections may have contributed to the superior memory for decision-point objects. Janzen et al. [37] and Miller et al. [40] controlled for potential factors such as viewing time and number of experienced viewpoints by having their participants passively transported through the virtual environment rather than actively navigating; in spite of equal viewing time for both types of objects, and only experiencing a single view of each object, they still saw evidence of encoding differences in both the fMRI and behavioural results.

To rule out the difference in number of experienced views or in viewing times as possible explanations of our decision-point effects in Condition B, we conducted a second experiment in which we showed participants videos of trajectories through a town instead of asking them to actively drive. As in Experiment 1, half the participants had one starting point and the other half had two.

### Experiment 2

In Experiment 2, participants watched videos showing a fixed route through the town. In Condition A participants saw the same route in both the forward and the reverse direction, while in Condition B they only saw the route in one direction, starting from a view facing Mike’s Restaurant and ending at a view of House of Pizza. As in Experiment 1, we interleaved blocks of study trials with blocks of memory test trials from two different tested viewpoints. Because participants’ trajectories through the town were highly constrained, relative to the free navigation conditions in Experiment 1, we were able to use much larger towns with more stores and objects while keeping the total study time to within a reasonable limit. Although active navigation might be more effective, we predicted that passively viewing a continuous trajectory through the town would still lead to the generation of a continuous cognitive map of the environment. Using a similar passive navigation paradigm and a recognition memory test, Janzen and van Turennout [37] found greater parahippocampal activity for decision-point objects even when the participants did not correctly recognize them. Thus, as in the previous two experiments, we predicted that objects at decision points would more likely be encoded as part of an allocentric cognitive map, and should therefore be remembered more consistently across the two tested viewpoints, particularly in Condition B (only one starting point).

#### Recognition accuracy

Recognition accuracy was better for decision-point objects. A two-way repeated measures Place x Condition ANOVA revealed a significant main effect of Place [*F* (1,48) = 4.917, *p* = 0.031], but no main effect of Condition [*F* (1,48) = 0.902, *p* = 0.347] and no interaction between Place and Condition [*F* (1,48) = 1.317, *p* = 0.257]. As in Experiment 1, recognition memory for objects at decision points (*mean* = 93.1%, *SE* = 0.012) was significantly more accurate than for objects at non-decision points (*mean* = 90.6%, *SE* = 0.011) across conditions.

#### Pointing latency

Pointing latencies was faster for decision-point objects. A three-way repeated measures Place x Condition x tested Viewpoint ANOVA revealed a significant main effect of Place [*F* (1,48) = 5.673, *p* = 0.021], but no main effects of tested Viewpoint [*F* (1,48) = 1.939, *p* = 0.17] or Condition [*F* (1,48) = 0.244, *p* = 0.623], and no interactions. Pointing latencies for objects at decision points (*mean* = 3.465, *SE* = 0.165) were significantly faster than those for objects at non-decision points (*mean* = 3.643, *SE* = 0.189) across conditions, although the effect was very small (*mean* difference of less than 0.2 seconds).

#### Pointing errors

Pointing errors were affected by viewpoint, but not by navigational relevance. A three-way repeated measures Place × Condition × tested Viewpoint ANOVA of the pointing errors revealed a significant interaction between tested Viewpoint and Condition [*F* (1,48) = 12.283, *p* = 0.001], but no other main effects or interactions. Thus in contrast to the results obtained in Experiment 1 under active navigation conditions, navigational relevance did not significantly affect pointing errors when participants engaged in passive navigation. To identify the source of the viewpoint by condition interaction in terms of pointing errors, two separate two-tailed paired sample *t*-tests were conducted for each condition (for controlling for multiple comparison, significant *p* value was 0.025). There was no difference in the pointing errors between the two tested viewpoints in Condition A (*t* = 2.116, *df* = 24, *p* = 0.045), but pointing errors made from the familiar Mike's Restaurant viewpoint were significantly smaller than those made from the House of Pizza viewpoint (*t* = −2.895, *df* = 24, *p* = 0.008). The results showed the viewpoint effect only in Condition B, but not in Condition A, see Table 1.

#### Pointing consistency: Standard deviations

**Figure 2:**
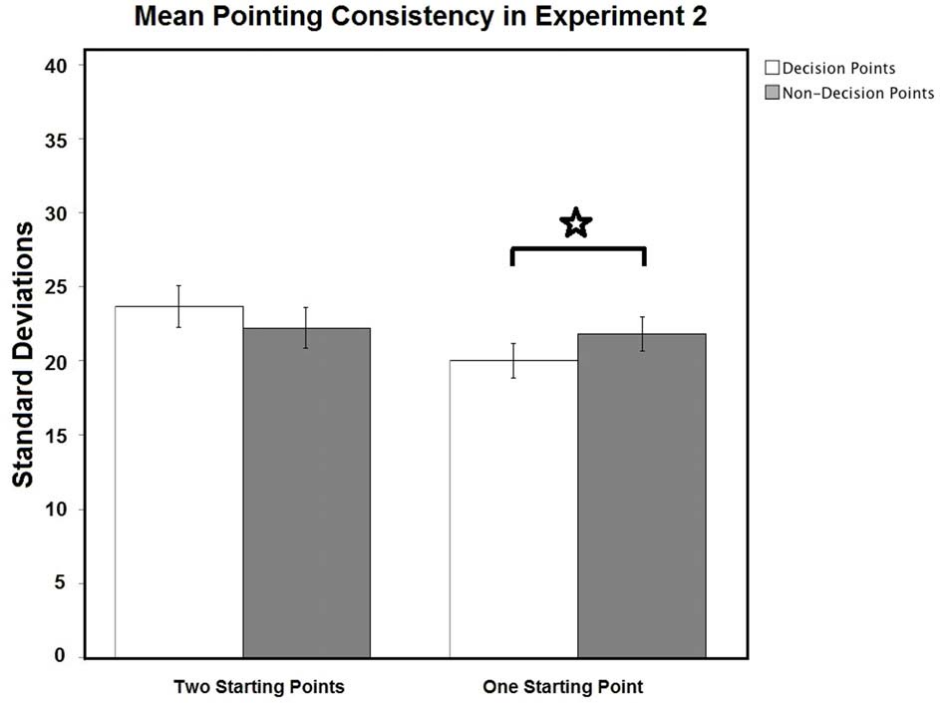
Pointing consistency in Experiment 2. Mean pointing consistency scores (95% confidence intervals) for decision and non-decision-point objects in Experiments 2-Passive navigation (two starting points vs. one starting point). White bar is for decision points and grey bar is for non-decision points. Pointing responses were more consistent for objects at decision points than for those at non-decision points in Experiment 2b (one starting point), but not in Experiment 2a (two starting points). ⋆ means p<0.05.

Navigational relevance affected pointing consistency when there was only one starting point. A two-way repeated measures Place × Condition ANOVA of the pointing consistency standard deviation scores revealed a significant interaction between Place and Condition [*F* (1,48) = 5.681, *p* = 0.021], but no main effects of Place [*F* (1,48) = 0.081, *p* = 0.777] or Condition [*F* (1,48) = 0.756, *p* = 0.389], see Figure 2. To investigate the interaction between Place and Condition in terms of pointing consistency, two-tailed paired sample *t*-tests were used. In Condition B, pointing responses were significantly more consistent for decision-point objects than for non-decision-point objects (*t* = −2.484, *df* = 24, *p* = 0.020), but no such difference in Condition A (*t* = 1.244, *df* = 24, *p* = 0.226). Note: for controlling for multiple comparisons, significant *p* value is 0.025. As in Experiment 1, reducing the number of starting points resulted in greater consistency of pointing errors across viewpoints for decision-point objects relative to non-decision-point objects, but there was no such difference when there were two starting points. There are two possible sources of the reduced variability in pointing errors to decision-point objects: the errors themselves could be smaller, and/or the errors could be more systematic across viewpoints. Our analysis of the pointing errors rules out the former interpretation, as there was no effect of navigational relevance on pointing error magnitude. Thus, the effect of navigational relevance on consistency, but not on accuracy, indicates that if an object was mis-localized when tested from one end of town, it tended to be mis-localized to the same (allocentric) direction when tested from the other end of town.

An alternative explanation for the consistency difference in Condition B could be alignment effects: some of our objects (3 decision-point objects and 2 non-decision-point objects) were viewed from directions aligned with the main longitudinal axis of the town and thus aligned with the tested viewpoints, whereas others (2 decision point and 3 non-decision-point objects) were viewed along the perpendicular axis. To rule out this alternative explanation, we performed the same analysis in Condition B on consistency scores for a subset of the objects, including two decision-point objects and two non-decision-point objects, which were pairwise matched for their average distances to the midline of the town, with one object of each type located on a part of the route aligned with the main longitudinal axis of the town and one object of each type located on a part of the route that was aligned with the perpendicular axis (two objects at far left of the town and two at the far right of the town, see EXPERIMENT 2 Materials for details). Only trials where there were pointing errors for both objects (one decision-point object and one non-decision-point object) in each pair were used in each block, and then averaged by object types and over blocks. One participant’s data were eliminated in this analysis due to unsuccessful recognition of all four objects over two blocks. We hypothesized a priori that even with this reduced set of responses to the matched pairs of objects, navigational relevance would still be a modulating factor, leading to greater pointing consistency for objects at decision points. A one-tailed paired sample *t*-test of the consistency scores revealed that, as with the full set of data, pointing responses for just these alignment-matched objects were significantly more consistent across viewpoints for decision-point objects (*mean* = 23.847, *SE* = 1.638) than for non-decision-point objects (*mean* = 27.877, *SE* = 2.492) (*t* = −1.958, *df* = 23, *p* = 0.0315). Although the effect was weakened by only analyzing less than half (8 out of 20) of the responses, the navigational effect was still significant.

The results of both Experiments 1 and 2 suggest that when participants have more limited experience with an environment (one starting point rather than two), objects at decision points are remembered more consistently, and are thus more likely to be encoded in a view-invariant manner. Janzen and van Turennout’s [37] findings of greater fMRI parahippocampal activation during recognition memory judgments for objects placed at T-junctions relative to L-junctions suggest that different encoding mechanisms may be employed for these two types of objects. However, they did not explicitly test spatial memory. Building on their results, we saw a difference in the consistency of spatial memory errors, as hypothesized, with the responses for objects at non-decision points showing less consistency across tested viewpoints in spite of similar pointing error magnitudes for the two types of objects. Unlike in Experiment 1, the total viewing time and number of experienced viewpoints for the two types of objects were held constant in Experiments 2. The greater consistency of pointing errors for decision-point objects, in spite of a lack of difference in average absolute pointing errors for these objects, means that even when participants could not accurately recall the correct locations of the decision-point objects, they mis-localized these objects in a manner that was consistent across the two tested viewpoints, whereas pointing to non-decision-point objects was no less error-prone but less consistent across viewpoints. This finding provides strong support for the hypothesis that decision-point objects were more likely to be encoded within an allocentric frame of reference.

Across both experiments, whether participants navigated freely or passively, when they were biased to have fewer spatial reference directions (one starting point rather than two), pointing errors were less consistent for non-decision-point objects compared to decision-point objects. This was true even when participants only saw objects from a single view (Experiment 2, Condition B), suggesting that for objects that are highly relevant to navigation, even exposure to a single view may be sufficient for their incorporation into an allocentric representation, whereas for objects less relevant to navigation, exposure from multiple viewpoints may be required.

Our original hypothesis was that objects could either be 1) treated as landmarks and incorporated within allocentric maps of space, or 2) encoded egocentrically. The object’s relevance to navigation and spatial cognition, rather than the amount of attention paid to the object, was hypothesized to be a critical factor in determining whether the allocentric spatial memory system is engaged in object encoding. To further investigate this possibility, we designed another experiment in which we manipulated explicitly the type of attention participants paid to objects.

### Experiment 3

In Experiment 3, we manipulated participants’ attention explicitly by asking half of them to pay particular attention to the appearance and the other half to attend to the locations of objects. We hypothesized that when attending to appearance, participants would encode objects simply as objects, not as landmarks. In this case, navigational relevance would not contribute to memory encoding, and they would be primarily engaging their object recognition system (associated more with the ventral visual pathway) to process the objects. On the other hand, asking participants to pay attention to the locations of the objects was hypothesized to engage visuo-spatial attention and navigation circuits associated with the dorsal visual stream (and more specifically, with the parieto-frontal and parieto-temporal branches of the dorsal stream [1]) to a greater degree, leading to the incorporation of the object into a configural, allocentric representation of space in the medial temporal lobe. Thus, we predicted that when attending to objects' appearance participants’ spatial memory would be equally accurate and consistent for decision and non-decision-point objects, whereas when attending to objects’ locations, the greater navigational relevance of decision-point objects would favor their encoding as landmarks within an allocentric framework, relative to non-decision-point objects. Moreover, we tested whether video game experience would have an effect on spatial memory or navigational strategies.

We hypothesized that when attention was directed toward objects’ appearance, the pointing consistency results we observed in the above experiments would disappear, and spatial memory would be less accurate for all objects, whereas when attention was directed toward objects’ locations, the decision-point effect would be enhanced compared to results in Experiment 1-Condition B.

#### Recognition accuracy

Recognition accuracy was better for decision-point objects. A two-way repeated measures ANOVA with Place (Decision vs. Non-Decision Points) as a within subject factor and Attention (Appearance vs. Location condition) as a between subject factor revealed a significant main effect of Place [*F* (1,55) = 4.348, *p* = 0.042], but no main effect of Attention [*F* (1,55) = 0.094, *p* = 0.76] and no interaction between Place and Attention [*F* (1,55) = 0.001, *p* = 0.981] on recognition memory accuracy. Recognition memory accuracy was better for objects at decision points than for those at non-decision points (Appearance: DP *mean* = 86.69%, *SE* = 0.03, NDP *mean* = 81.17%, *SE* = 0.029; Location: DP *mean* = 87.64%; *SE* = 0.031, NDP *mean* = 82.25%, *SE* = 0.029).

#### Pointing latency

Pointing latency was faster at the familiar viewpoint. A three way repeated measures Place x Attention x tested Viewpoint ANOVA revealed a significant main effect of tested Viewpoint [*F* (1,55) = 4.224, *p* = 0.045] on pointing latency, but no other significant main effects or interactions. Pointing responses were faster when tested from the House of Pizza viewpoint (the starting point) than the Mike’s Restaurant viewpoint for both types of object locations and both attention conditions (House of Pizza *mean* = 4.804, *SE* = 0.291; Mike’s Restaurant *mean* = 5.487, *SE* = 0.476).

#### Pointing errors

Pointing errors were smaller at the familiar viewpoint. A three-way repeated measures Place x Attention x tested Viewpoint ANOVA showed that there was a significant main effect of tested Viewpoint [*F* (1,55) = 5.204, *p* = 0.026] on pointing errors, but no other significant main effects or interactions. Pointing errors were smaller at the more familiar House of Pizza viewpoint than at the Mike’s Restaurant viewpoint for both types of object locations and both attention conditions, see Table 1 for means and SEs.

#### Pointing consistency: standard deviations

**Figure 3:**
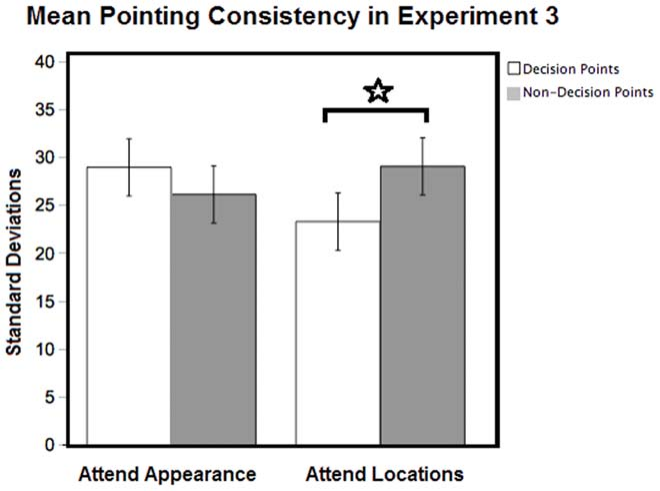
Figure 3. Pointing consistency in Experiment 3. Mean pointing consistency scores (95% confidence intervals) for decision and non-decision-point objects in Experiments 3- Active navigation with attention manipulation (attend Appearance vs. attend Locations). White bar is for decision points and grey bar is for non-decision points. Pointing responses were more consistent for objects at decision points than for those at non-decision points in the Location condition, but not in the Appearance condition. ⋆ means *p*<0.05.

Pointing responses were more consistent for objects at decision points in the Location_condition, but not in the Appearance condition. A two-way repeated measures Place x Attention ANOVA revealed a significant interaction between Place and Attention [*F* (1,55) = 5.156, *p* = 0.027], but no main effects of Place [*F* (1,55) = 0.605, *p* = 0.44] or Attention [*F* (1,55) = 0.135, *p* = 0.715] on pointing consistency. Based on the results of our previous experiments, we predicted a priori that pointing consistency would be worse for objects at non-decision points than for those at decision points in the Location condition. To test this prediction, we therefore used a one-tailed paired sample *t*-test, which revealed that pointing scores were significantly more consistent for objects at decision points versus non-decision points in the Location condition (*t* = −2.186, *df* = 27, *p* = 0.019, DP *mean* = 23.457, *SE* = 2.424; NDP *mean* = 29.2486, *SE* = 3.461), but not in the Appearance condition (*t* = 1.043, *df* = 28, *p* = 0.153, DP *mean* = 29.1428, *SE* = 2.382; NDP *mean* = 26.3072, *SE* = 3.401), see Figure 3. (Note: for controlling for multiple comparisons, significant *p* value is 0.025).

#### Navigation efficiency

**Figure 4:**
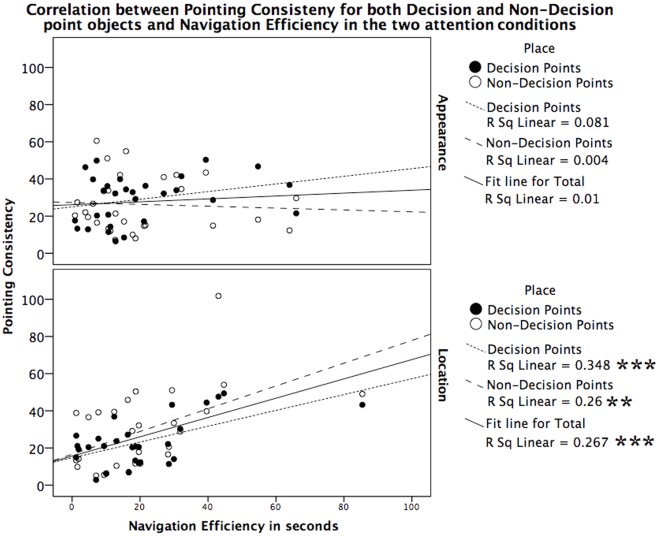
Figure 4. Correlation between navigation efficiency and pointing consistency in Experiment 3. Correlation between navigation efficiency and pointing consistency for decision and non-decision-point objects for both attention conditions in Experiments 3. Black dot is for decision points and white dot is for non-decision points. There are significant positive correlations between navigation efficiency and pointing consistency for decision-points and non-decision-point objects in the Location condition, but not in the Appearance condition. The more consistent the pointing responses made from two tested viewpoints, the more efficient the participants were in delivering passengers. **means *p*<0.01, ***means *p*<0.001.

Pointing consistency was significantly correlated with navigational efficiency. An analysis of the correlation between participants’ navigation efficiency and pointing consistency revealed a positive correlation for both decision-point objects [*r* (28) = 0.59, *p* = 0.001] and non-decision-point objects [*r* (28) = 0.51, *p* = 0.006] in the Location condition, but no such correlation in the Appearance condition (DPs: [*r* (29) = 0.284, *p* = 0.136]; NDPs: [*r* (28) =  −0.06, *p* = 0.757]), see Figure 4. This result suggests that in the Location condition, objects were encoded as landmarks and facilitated efficient navigation, while in the Appearance condition they were not.

#### Questionnaire results

Video Game players were more efficient at navigation, and more accurate but no more consistent in pointing. There were 32 participants who self-identified as video game players (17 in the Appearance condition) and 25 who did not (12 in the Appearance condition). Separate two-way repeated measures Place x Video Game Experience ANOVAs were conducted on pointing latency, pointing errors and consistency. There was a significant main effect of video game experience on pointing errors [*F* (1,55) = 6.581 *p* = 0.013], but no other significant main effect or interaction with any other measure. Video game players had significantly smaller pointing errors (*mean* = 27.602, *SE* = 2.757) than non-players (*mean* = 38.279; *SE* = 3.119). However, video game experience was not a significant factor in pointing latency or pointing consistency.

We conjectured that video gamers might be more accurate at pointing to objects, even though they were no more consistent in the errors they made across viewpoints, because of their superior ability to navigate and encode routes, and subsequently to recall and/or imagine specific routes in the town. A two-tailed independent *t*-test to compare navigational efficiency of gamers to that of non-gamers revealed that participants who played video games (*mean* = 16.2519) were also more efficient in delivering passengers to their destinations than non-players (*mean* = 25.9189) (*t* = −2.125, *df* = 55, *p* = 0.038).

Much research has been devoted to the roles of the dorsal and ventral visual pathways, commonly referred to as the “what and where”, “what and how to”, or “perception and action” pathways (see e.g. [1]–[3]). However, there have been relatively few attempts to manipulate the degree to which objects are processed by one pathway or the other within a single study. While we did not measure directly what neural circuits were involved, in Experiment 3 we manipulated the type of attention participants paid to objects, in an attempt to bias them in favor of either the object processing stream or the visuo-spatial stream. The results of Experiment 3 support our hypothesis that directing participants’ attention to appearance vs. location affected how the objects were encoded. When asked to attend to the appearance of the objects, participants did not show any differences in pointing consistency between decision-point and non-decision-point objects. We suggest that this is because the objects were not treated as landmarks; therefore, navigational relevance would not contribute to memory encoding. On the other hand, asking participants to pay attention to the locations of the objects encouraged them to treat the objects as landmarks, not just simply as objects. This type of processing is postulated to engage the dorsal visual pathway, both the parieto-prefrontal branch for top-down executive control of visuospatial processing and the parieto-medial temporal branch for encoding within a world-centred reference frame [1], leading to the incorporation of the object into a configural, allocentric representation of space in the medial temporal lobe. Consistent with this prediction, pointing responses were more view-invariant for decision-point objects than for non-decision-point objects when attention was directed to objects’ locations, but not when attention was directed toward objects’ appearances.

It is somewhat surprising that our attention manipulation did not produce any main effects on recognition memory, pointing latency or pointing errors. It could be that both attention conditions resulted in equally strong, but qualitatively different attentional resources being devoted to the objects in the two attention conditions. The differential effect of the attentional manipulation on pointing consistency supports this notion, but further experiments are required to demonstrate that distinctly different neural circuits were recruited in the two conditions.

Chun and Jiang [44] suggested that memory for context could be implicitly learned and used to guide spatial attention for detecting the target among distractors. We suggest that without an explicit attentional manipulation, people might automatically pay attention to the locations of objects or building that are relevant for navigation in everyday life; this could explain the decision-point effect shown in our first two experiments. When attention was manipulated explicitly toward the objects’ locations, this decision-point effect was enhanced, whereas when attention was focused on the objects’ appearance, the effect was eliminated.

Both decision and non-decision points benefit from aligning the tested viewpoint with a salient reference direction, as the pointing responses were faster and more accurate when tested from House of Pizza viewpoint, the starting point, than from Mike’s Restaurant. However, the analysis of pointing consistency revealed that spatial memory was less affected by viewpoint changes for decision-point objects than for non-decision-point objects.

An interesting double dissociation is apparent in the results of Experiment 3: video game experience was associated with faster and more accurate pointing responses and greater navigation efficiency but no greater pointing consistency. In contrast, the attentional manipulation affected pointing consistency but not pointing accuracy or latency. Further experiments would be required to tease apart what systems or strategies are at play that could explain these differences. One possibility is that gamers are more adept at employing egocentric route recall strategies, whereas attention to location versus appearance causes a (within-subjects) processing switch between spatial and non-spatial object encoding systems.

## Discussion

The results of our three experiments are consistent with our prediction that navigational relevance contributes to whether objects are encoded as landmarks within an allocentric framework. It is important to note that allocentric encoding might not be unique to the dorsal visual pathway; objects might individually be encoded in a view-invariant manner within the ventral visual pathway (the so-called “what pathway”) (for more recent interpretations of the role of the ventral visual stream, see e.g. [45], [46]). However, this type of allocentric or view-invariant coding of individual objects is distinctly different from the notion of allocentric spatial coding of a conjunction of the objects and features within a large-scale environment into a “cognitive map”, as typically attributed to the hippocampus. It is the latter type of allocentric encoding that we focus on in the present experiments.

Our novel pointing consistency measure proved to be sensitive to the navigational relevance manipulation, across all three experiments, in both active and passive navigation conditions. This greater viewpoint-invariance in memory for objects at decision points was modulated by whether participants began their navigation from both ends of town or just one (Condition A vs. B in Experiments 1 and 2), and whether participants attended to the objects’ locations or appearance (Experiment 3). When participants only began navigating from one end of the town, making one tested viewpoint more accessible than the other, spatial memory from the less familiar viewpoint was more disrupted for objects that were not at decision points. Similarly, when attention was explicitly directed toward the objects’ locations, memory for objects at decision points was even more consistent across tested viewpoints. Even when participants were no more accurate at pointing to decision-point objects (Experiments 2 and 3) they were still more consistent across tested viewpoints for objects at decision points relative to other objects. Taken together, our results suggest that when people process objects in the service of navigation, and when they are exposed to multiple views of objects, both factors contribute to the encoding of objects within their broader spatial context as allocentric spatial maps. These results are broadly consistent with the framework of the BBB model [32], which proposes that a landmark’s visual attributes are processed within the ventral visual stream, its spatial attributes are processed within the dorsal visual stream, and both are integrated into a large-scale spatial representation within the medial temporal lobe. Here we suggest a further refinement of the BBB theory, that is, objects in the environment may or may not be treated as landmarks, according to where they are located and how they are attended to.

If navigational relevance leads to more view-invariant location memory, does this necessarily imply allocentric coding? An alternative interpretation is that navigational relevance improves spatial encoding within an egocentric memory system. However, our results argued against this interpretation. If memory is allocentric, then reducing the number of viewpoints experienced at study should have little impact on the variability of pointing errors across tested viewpoints. As predicted, the manipulation of reducing the number of starting points in Experiments 1 and 2 only affected the consistency in pointing errors for non-decision-point objects, but not for decision-point objects. Thus, the results of our experiments are consistent with our prediction that objects at decision points were more likely than other objects to be encoded allocentrically. Moreover, it may only require exposure to a single viewpoint (Condition B in Experiment 2) to generate an allocentric representation of an object that is navigationally relevant.

While the effect of navigational relevance on viewpoint invariance was consistent across all three experiments, its effect on pointing accuracy versus latency differed. When there was a single starting point, pointing responses to objects at decision points were more accurate but no faster in Experiment 1 (active navigation), faster but no more accurate in Experiment 2 (passive navigation), and neither faster nor more accurate in Experiment 3. While participants in Experiment 3 only had a single block of study and test trials, those in Experiments 1 and 2 had multiple interleaved study-test blocks, affording the opportunity to develop different strategies over blocks on the pointing task in the active versus passive navigation conditions. There are several different strategies that could be used, including 1) employing an allocentric representation, 2) recalling multiple view-based snapshot memories and judging the alignment of the test view with the stored snapshots, 3) mentally rotating a single stored view of the scene to match the test view, or 4) imagining navigating from the tested view to the experienced view. Strategies 3 and 4 should result in longer reaction times relative to the strategies 1 and 2, while strategy 2 would be less accurate than an allocentric strategy, particularly when fewer stored viewpoints are available, as in the single starting point condition. In the passive navigation experiment in Condition B where only a single view of each object was seen, strategy 2 would be infeasible, but either strategy 3 (mental rotation of a stored view) or 4 (route recall/mental navigation) could have been employed. Adopting either of these egocentric strategies for non-decision-point objects and an allocentric strategy for decision-point objects would explain the observed reaction time differences. On the other hand, the active navigation conditions of Experiment 1 permitted participants to approach each object from multiple directions and via multiple routes. This might bias participants to favor strategy 2, attempting to match the test viewpoint to multiple viewpoint-specific snapshot memories; such a strategy would be less accurate than an allocentric one, and could explain the lower accuracy for non-decision-point objects. Future research is required to determine which if any of the strategies discussed here might be employed, and under what conditions, to cause the observed differences between active and passive navigation.

Unexpectedly, video game experience was associated with greater pointing accuracy and greater navigational efficiency, but no greater pointing consistency. One reason for this pattern of results could be that the advantage conferred by video game experience in our task is due to better egocentric encoding and recall of routes rather than superior allocentric encoding of objects in their spatial context. While individual differences in encoding and retrieval strategies were not the main focus of the present experiments, there is a growing literature on spatial navigation supporting the notion that individuals do tend to favor either an allocentric strategy or an associative response strategy, each of which is associated with its own distinct neural circuits (see e.g. [27], [47]). Moreover, preferential use of either the former or the latter strategy is associated with corresponding grey matter differences in the hippocampus versus basal ganglia [47]. Future studies could probe in greater detail what strategy participants were employing in the tasks studied here, and how individual differences may contribute to when objects are incorporated into cognitive maps.

While the experiments reported here focused on how objects are encoded within large-scale spaces, other studies have identified additional factors at play in smaller spaces where the collection of objects can be viewed simultaneously. Mou, McNamara and colleagues proposed that the interobject relations form an intrinsic reference system that contributes to long-term spatial memory [25], [34]–[36], [48]. When intrinsic structure (object defined) and extrinsic structure (environmental defined) are congruent, they jointly define the reference direction of spatial memory, while when they conflict, the first learning perspective (egocentric experience) defines the reference direction of spatial memory [49]. Having a preferred reference direction for accessing spatial memory is not inconsistent with the use of an allocentric representation. As predicted by the BBB model, an access cue such as a view of a specific landmark arrives as an egocentric sensory input pattern, and must first be transformed into an allocentric representation, and then subjected to an associative recall process to retrieve a complete allocentric spatial memory. Consistent with the preferred reference direction findings of Mou and colleagues [25], [34]–[36], [48], we observed a viewpoint familiarity effect across all three experiments when participants started navigation from one end of town.

Our findings suggest that people may switch flexibly between egocentric and allocentric representations, according to the type of attention paid to objects. However, there are a number of limitations to the present set of experiments that warrant further study. First, we focused on within-subject encoding differences for objects at different locations, but we did not assess in detail potential between-subject strategy differences. The latter may be a product of both short-term context and long-term experience. For example, our results hinted at strategic differences between video gamers and non-gamers, and between participants engaged in active versus passive navigation. Future studies could investigate in greater detail the basis of such individual differences, with additional measures of spatial strategy use including questionnaires, secondary allocentric tasks such as navigation with detours or short-cuts, and fMRI to determine whether distinct neural circuits are recruited. Moreover, we focused on objects that are relatively small and contained within the confines of the larger space, but more profound encoding differences might be seen with larger distal cues. Finally, there is a growing literature on the encoding of large-scale spaces at multiple spatial scales. Individual objects might be encoded differently at multiple spatial scales when they are clustered in different regions of space, creating both inter-object relations at a local scale, and object-environment relations at a global scale.

## Methods

### Experiment 1

#### Participants

Sixty McMaster University students ranging in age from 18 to 25 years (mean age 19.63) participated in the experiment. There were 30 participants in each condition (12 males and 18 females in Condition A; 13 males and 17 females in Condition B). Participants had normal or corrected-to normal vision, and received partial course credit or $10 for taking part in the experiment. This study was reviewed and approved by the McMaster University Research Ethics Board. Written informed consent was obtained from all participants involved in this study.

#### Materials

**Figure 5:**
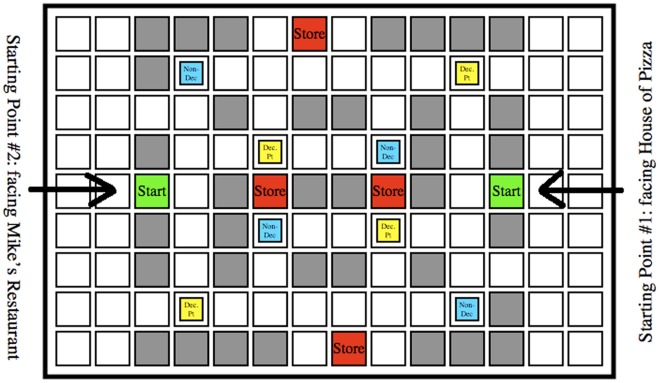
Figure 5. Virtual town used in Experiments 1 and 3. Town’s layout (14 by 9 VR units in size) used in Experiments 1 and 3. The grey squares are non-distinctive uniformly textured buildings at locations where the participants are not able to drive into. The “Store” squares are the stores that serve as passenger drop-off locations. The two “Start” squares are the two starting points, locating at either end of the town (Mike’s Restaurant and House of Pizza). The “Non-Dec” squares are the non-decision points where the objects were placed; at these locations participants can only turn in one direction. The “Dec. Pt.” squares are the decision points where the objects were placed; at these locations participants can either turn left or right. All the white squares indicate locations along the routes that participants can navigate in the town. All the objects are located in the middle of the street; participants can go around the objects.

**Figure 6:**
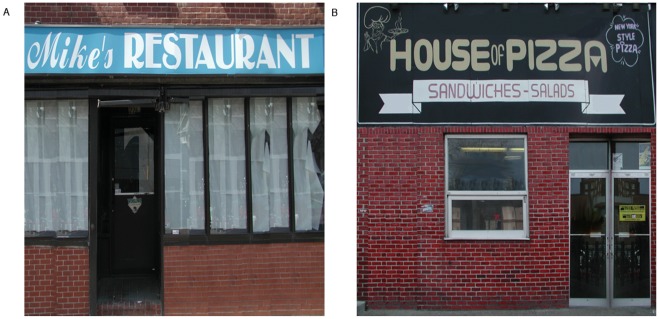
Two starting points (also the tested viewpoints): (A) Mike’s Restaurant and (B) House of Pizza.

**Figure 7:**
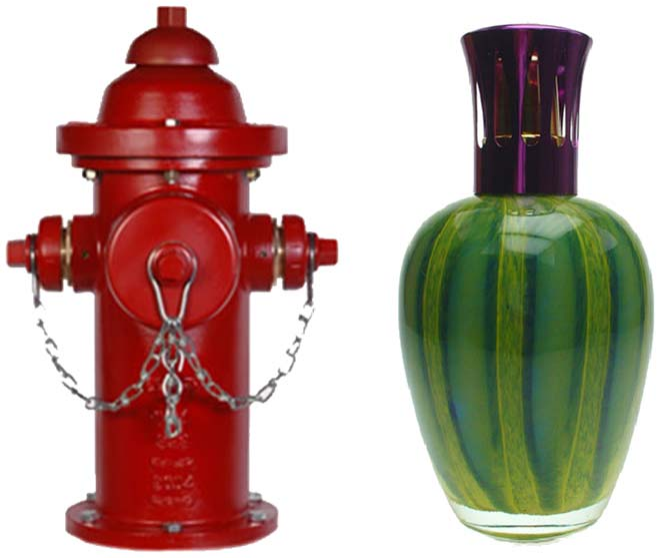
Sample objects used in the towns.

We employed Kahana’s “Yellow Cab” virtual driving simulator (see http://memory.psych.upenn.edu/Research) for constructing the environment and for simulating the virtual taxi game for the study phase of the experiment. There were two rectangular shaped towns (14 by 9 VR units in size) with different layouts (see Figure 5). Each participant experienced only one of the two towns. In each town, there were two distinctive buildings, Mike’s Restaurant (see Figure 6A) and House of Pizza (see Figure 6B), respectively located at the two ends of the town, which marked the two alternative starting points for each passenger pickup and also the two tested viewpoints for the spatial memory tests. There were four stores designated as passenger drop-off or goal locations. The two starting points were not used as drop off locations. There were 19 objects (see Figure 7) used in the experiment, 8 of which appeared in the town and the remaining eleven of which were only shown at the object pre-exposure phase and served as distractors for the subsequent recognition memory task. Four of the 8 objects in the town were placed at decision points (T-shaped intersections), where the participants could decide to turn right, turn left, or continue straight. The other four objects were placed at non-decision points (L-shaped intersections), where the participants could only turn in one direction. The locations of decision-point and non-decision-point objects were matched pairwise with respect to their distance from the town midline and distance from the town end-point, that is, with the viewer at location (0,0), for every decision-point object at location (x, y) there was a corresponding non-decision-point object at location (−x, y) (see Figure 5). Thus, during the pointing task with the participant placed at either end of the town, the average distance of objects to the town midline and to the observer was equal for the two groups of the objects. The locations of individual objects in each town remained constant across blocks. During the study phase, participants used either the joystick or the arrow keys on the keyboard to control their navigation, allowing them to turn in any direction, control their speed, or do a U-turn.

**Figure 8:**
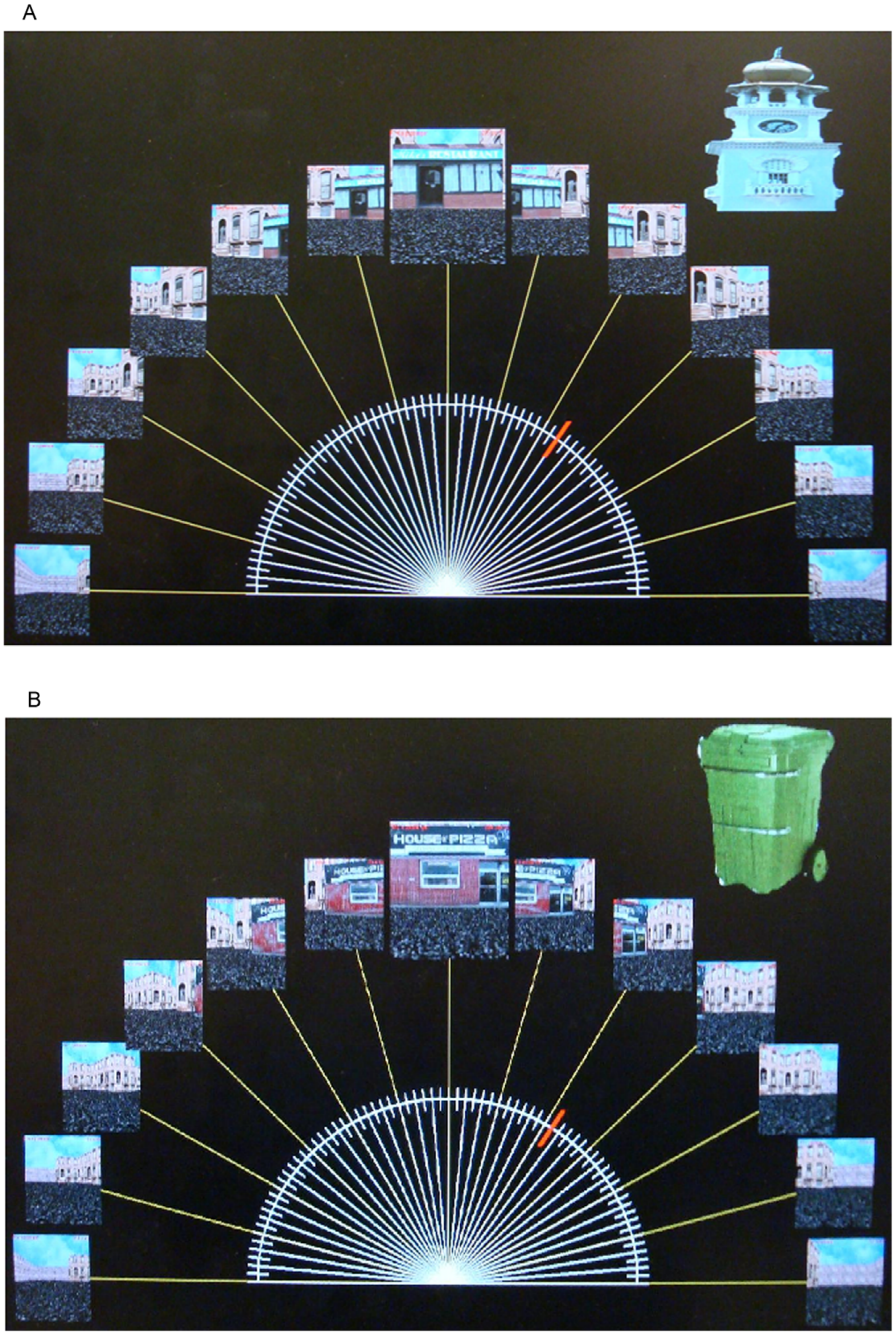
Figure 8. Pointing task: two testing viewpoints. The navigators used in the pointing task. In a semi-circular arc along the top of the navigator, pictures of different views of the actual town are shown, as seen from different angles at the starting location at either one end or the other end of the town. At the tip of the compass pointer (red) an image was shown of the target object for the current trial. It could be moved by moving the pointer. A. Navigator from Mike’s Restaurant point of view; B. Navigator from House of Pizza point of view.

The memory test was implemented in Matlab with the Psychophysics Toolbox extensions [50], [51]. On each memory test trial, there was a half compass shaped figure (a navigator) with pictures of multiple views of the actual town seen from either one end or the other end of the town on the top of the navigator (see Figure 8). Images of different views of the two were shown at the ends of the compass lines, so that the view from straight ahead was located at the top of the compass (forward direction), the views when looking to the left to varying degrees were located at corresponding points to the left of forward, etc. Additionally, at the tip of the compass pointer an image of the target object for the current trial appeared; this target object moved with the pointer. The target was always one of the 19 objects (8 of which had been in the town and 11 of which were distractors), and the participant could use the mouse to rotate the compass pointer (along with the target) to point in the remembered direction of the target object from the displayed viewpoint and make a mouse-click to indicate a pointing response or press the space bar to indicate that an object was not recognized as having been in the town.

#### Procedure

There was a total of four blocks, each consisting of an object pre-exposure, a study and a test phase. In the object pre-exposure phase, each of the 19 objects appeared for two seconds followed by a blank screen for one second. This established some degree of familiarity of the distractor objects so that the subsequent recognition memory task would be more challenging. In each study phase, the participant was asked to act as a taxi driver whose task was to roam around and find passengers and deliver them to specific locations (i.e., stores). A trial began with the participant located at one of the two ends of the town, facing toward the middle of the town, and he/she was asked to freely navigate until a passenger was found and “collected” by bumping into the passenger. A textual cue then appeared, e.g. “Please take me to the Flower Patch store, I will give you 100 points”, and the participant was instructed to navigate as quickly and efficiently as possible to drop off the passenger to the goal location by bumping into it. In Condition A, each time the participant dropped off a passenger, he or she was re-located to the opposite end of the town from the previous trial, facing either Mike’s Restaurant or House of Pizza, before being cued to collect the next passenger. In Condition B, participants were always relocated to House of Pizza after each pick up. There were five passenger deliveries in each of the four blocks, hence a total of 20 passenger deliveries. The participant’s location and viewing direction were recorded every 30–40 ms throughout the entire study phase. The memory test combined simultaneous tests of recognition memory and spatial memory. On each trial, if the participant thought the object had not appeared in the town, he or she pressed the ‘space bar’, and the next object would be displayed. Otherwise, he or she then pointed in the direction of the object’s remembered location from the displayed viewpoint (see Figure 8) by using the mouse to move the compass pointer to the desired direction, and then pressing the left mouse button. We did not measure recognition memory reaction time separately, but we did measure pointing latency, as our memory test combined recognition and spatial memory. For both conditions A and B, in each memory test phase, the participant had to respond to each object twice, once from each end of the town (see Figure 8). The recognition responses, pointing directions and total reaction time for the combined spatial/recognition memory response were recorded during the memory test phase.

#### Data analysis

In McNamara et al’s experiments, a gender effect was not consistent: in most of their studies, there was no gender effect (e.g. [4], [25], [34], [42], [49], [52] and in other studies, males were more accurate [43] or faster [35] than females. Moreover, Lavenex and Lavenex [53] did not find a gender effect on spatial relational learning. Gender is not a focus in our study here and we did not find a gender effect in our measurements. Therefore, we did not include gender as a factor in our analyses here.

Bonferroni correction was used for all the multiple comparisons throughout this paper. Loftus and Masson’s [54] method was used to calculate 95% confidence intervals shown in the figures.

Recognition Accuracy: The accuracy of the participants’ recognition memory was calculated as follows: If the participants indicated that they had seen the object, but the object was not used in the town, the response was counted as a false positive. If the participant indicated that they had not seen the object, but the object was used in the town, the response was counted as a false negative. Otherwise, the response was counted as a correct recognition. We calculated percent correct recognition separately for objects at decision points and those at non-decision points, averaged across blocks. We then compared the difference in recognition accuracy between decision and non-decision points in Conditions A and B by using a two way repeated measures Place x Condition ANOVA.

Pointing Latency: There were two reaction time scores (in seconds) for each object in each block: one for each of the two tested viewpoints. We averaged the reaction time over correct responses across blocks for objects at decision points for each viewpoint, and did the same for those at non-decision points to get pointing latency for decision and non-decision points for each of the two tested viewpoints. We compared the pointing latencies using a three-way repeated measures ANOVA, with Place (decision vs. non-decision points) and tested Viewpoint (Mike’s Restaurant vs. House of Pizza) as within-subject factors and Condition (one vs. two starting points) as a between-subject factor.

Pointing Errors (Average Absolute Pointing Errors): In each block, participants had to point to each object once from each of the two starting points. Therefore, each participant had two pointing responses for each object, one from the viewpoint of Mike’s Restaurant and one from the viewpoint of House of Pizza. A pointing error was defined as the signed value, in degrees, of the difference between the pointing response and the object’s actual direction (participant’s response in degrees minus the object’s correct direction in degrees). Therefore, for each starting location we have four raw signed pointing errors for decision-point objects and four for the non-decision-point objects for each participant in each block if the participants correctly recognized all the objects used in the town. The signs of the pointing errors from one end of town were reversed so that consistent spatial memory errors for the same object from the two viewpoints would have the same sign; for example, a pointing error of ten degrees clockwise from Mike’s Restaurant and ten degrees counterclockwise from House of Pizza (the opposite end of town), after this sign change correction, would be coded equivalently as signed errors of +10.

We calculated the absolute value of all the raw signed pointing errors for each participant across blocks for decision points and similarly for the non-decision points. Thus, each participant had two average absolute pointing errors for each of the two tested viewpoints, one for decision points and one for non-decision points. We used a three -way repeated measures ANOVA to compare differences in pointing error between decision and non-decision points and between the two tested viewpoints in the two conditions (one vs. two starting points).

Pointing Consistency (Standard Deviations): To test the hypothesis that participants were more likely to incorporate decision-point objects into an allocentric map of space, we developed a measure of viewpoint consistency in pointing errors. We reasoned that if a participant is using an allocentric representation of an environment to recall an object’s location, their pointing errors for that object should be consistent across the tested viewpoints, regardless of overall magnitude. Thus, if an objects’ location is remembered accurately from one viewpoint, it should be equally accurately remembered when tested from the other viewpoint. On the other hand, if an object is remembered incorrectly, resulting in a high pointing error from one viewpoint, the participant should make an error of the same magnitude but opposite sign when tested from the opposite viewpoint. In contrast, if s/he has an egocentric representation of an object’s location from a given direction within an environment, the pointing errors made between familiar and unfamiliar viewpoints would be more variable, because the participant may have to mentally rotate the representation in order to align it with the familiar stored viewpoint. We used the standard deviation of pointing responses (signed pointing errors) across the two tested viewpoints as a measure of the consistency of the pointing responses. If, for example, a participant consistently mis-located an object as being 10 degrees clockwise when tested from the Mike’s Restaurant viewpoint and 10 degrees counterclockwise when tested from the House of Pizza viewpoint, the signed errors for this object would both be +10 and the standard deviation across the two viewpoints would be zero. Note that we counted pointing errors in the clockwise direction from the Mike’s Restaurant viewpoint and counterclockwise from the House of Pizza viewpoint as both positive errors, while counterclockwise errors from Mike’s and clockwise errors from House of Pizza were counted as negative errors, see Pointing Error (Average Absolute Pointing Errors) for details.

The pointing consistency across the two tested viewpoints was calculated by taking the standard deviation of the two signed pointing errors that the participant made for each decision-point object from the two ends of the town, and then averaging these standard deviation scores across objects and across blocks at decision points, and similarly averaging those at non-decision points. Any object with less than two signed pointing errors was dropped from the consistency analysis. We thereby obtained two average pointing consistency scores for each participant, one for decision-point objects and one for non-decision-point objects. We analyzed these scores with a two-way repeated measures ANOVA (Place x Condition) to test the hypothesis that pointing responses across two tested viewpoints would be more consistent for objects at decision points than for other objects, particularly when there was only one starting point.

View Time: To assess whether participants spent more time viewing objects at decision points than viewing other objects during study, we calculated the “view time” of each object, for each block in each study phase, as the percentage of the total time that participants spent at locations where their facing direction placed the object within their field of view, and then calculated average percentage view times for the two types of objects. We averaged these scores across blocks and then used a two-tailed paired sample t-test to assess differences in view time.

### Experiment 2

#### Participants

Fifty McMaster University students ranging in age from 18 to 38 years (*mean* 20.44) participated in the experiment. There were 25 participants in each condition (8 males and 17 females in Condition A and 7 males and 18 females in Condition B). Participants had normal or corrected-to normal visions and received either partial course credit or $10 for taking part in the experiment. This study was reviewed and approved by the McMaster University Research Ethics Board. Written informed consent was obtained from all participants involved in this study.

#### Materials

**Figure 9:**
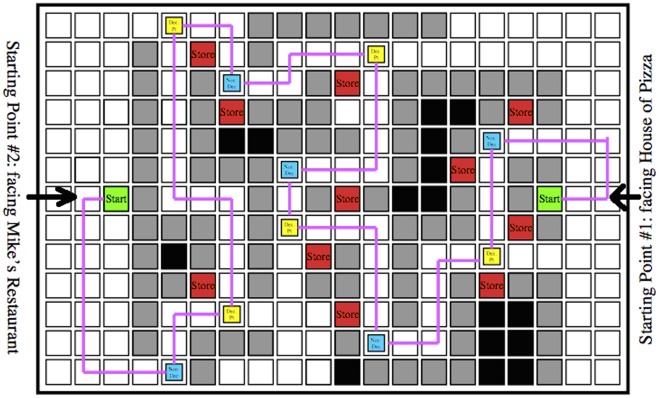
Figure 9. Virtual town used in Experiment 2. Town’s layout (20 by 13 VR units in size) used in Experiments 2, similar to the one used in Experiment 1, but larger. The black squares are places not visible to the participants. The pink line is the travel trajectory that participants watched in the video clips.

As in the previous experiment, we used Yellow Cab to create a rectangular town (20 by 13 VR units in size), see Figure 9. Although the layout of the town was different than those used in Experiment 1, we imposed the same constraint on the locations of the decision-point and non-decision-point objects, namely, they were equally distributed about the town midline (see Figure 9), so that the average distance from each object to the town midline was equal for the two groups of objects. There were 20 objects, all of which were used in the pre-exposure phase and subsequent recognition memory test, and 10 of which were located in the virtual town during the study/navigation phase, five at decision points and five at non-decision points. Two video clips were created by recording the experimenter driving in the town following the route shown in Figure 9, in which each time an object was approached, there was a turn in the route; one going from Mike’s Restaurant to House of Pizza, and the other traversing the reverse route from House of Pizza to Mike’s Restaurant.

#### Procedure

There were six blocks of trials, each including an object pre-exposure phase, a study phase and a test phase, as in Experiment 1. In the study phase, participants in Condition A watched both videos alternatingly three times each, and participants in Condition B watched video 1 six times. Prior to each study phase, participants were shown the rectangular outline of the town with Mike’s Restaurant and House of Pizza marked at each end, and told that they would be tested for their spatial memory of the objects after each block, and that they would be asked to draw a map of the layout of the town with all of the objects in it at the end of the experiment, to encourage participants to pay attention to the layout of the town during the study phase. The spatial memory test phase was the same as in Experiment 1.

#### Data analysis

Recognition accuracy, pointing latency, pointing errors, and pointing consistency were calculated and analyzed as in Experiment 1, except that we had five decision-point and five non-decision-point objects in each town rather than 4 of each object type.

### Experiment 3

#### Participants

Sixty McMaster University students participated in the experiment. Three participants whose recognition memory accuracy was less than 25% were excluded from the final data analysis. Therefore, there were fifty-seven participants; age ranged from 19 to 29 years, and the mean was 20.64. There were 29 participants (22 females and 7 males) in the Appearance condition and 28 (20 females and 8 males) in the Location condition. Participants received partial course credit or $10 for taking part in the experiment. This study was reviewed and approved by the McMaster Research Ethics Board. Written informed consent was obtained from all participants involved in this study.

#### Materials

The same materials were used as in Experiment 1.

#### Procedure

We used the same procedure as that used in Experiment 1 (active navigation) Condition B (single starting point) with the following changes.

At the beginning of the experiment, the participants were pseudo-randomly assigned to one of two attention conditions, either Appearance or Location, and were respectively asked in advance to pay particular attention to either the appearance or the locations of objects. They were told that their memory for the objects would be tested at the end of the experiment, and they would either have to recall as many visual details as possible of the objects in the appearance condition, or they would be asked to map out the locations of objects on a piece of paper in the location condition.

Whereas our previous experiments incorporated multiple blocks of interleaved study and test phases, in this experiment there was only one block of trials, including a single study phase and single test phase, in order to discourage participants from switching their attentional focus more towards the locations of objects after undergoing the first spatial memory test. The study phase was terminated once participants had found and delivered ten successive passengers or had reached the cutoff time of 35 minutes.

After the study phase and the pointing task, participants were asked: Do you play video games?

#### Data analysis

Participants’ recognition memory accuracy, pointing latency, pointing errors and pointing consistency across viewpoints were calculated as in the previous experiments. Additionally, we calculated the average navigation efficiency for each participant.

Navigation Efficiency: We subtracted the optimal time for each delivery based on the shortest route between the pick-up location and the destination from the actual time the participants took to deliver each passenger after the first 5 minutes navigating in the town. Hence, if the participant chose the shortest route to deliver the passenger, their efficiency score for this delivery would be zero. The first 5 minutes navigation was excluded from the analysis assuming participants used this time to learn the layout of the town.
